# Neuronal Cell Differentiation of Human Dental Pulp Stem Cells on Synthetic Polymeric Surfaces Coated With ECM Proteins

**DOI:** 10.3389/fcell.2022.893241

**Published:** 2022-06-14

**Authors:** Yan Gao, Zeyu Tian, Qian Liu, Ting Wang, Lee-Kiat Ban, Henry Hsin-Chung Lee, Akihiro Umezawa, Abdulrahman I. Almansour, Natarajan Arumugam, Raju Suresh Kumar, Qingsong Ye, Akon Higuchi, Hao Chen, Tzu-Cheng Sung

**Affiliations:** ^1^ School of Biomedical Engineering, The Eye Hospital of Wenzhou Medical University, Wenzhou Medical University, Wenzhou, China; ^2^ Department of Surgery, Hsinchu Cathay General Hospital, Hsinchu, Taiwan; ^3^ Graduate Institute of Translational and Interdisciplinary Medicine, National Central University, Taoyuan, Taiwan; ^4^ Department of Reproduction, National Center for Child Health and Development, Tokyo, Japan; ^5^ Department of Chemistry, College of Sciences, King Saud University, Riyadh, Saudi Arabia; ^6^ Center of Regenerative Medicine, Renmin Hospital of Wuhan University, Wuhan, China; ^7^ School and Hospital of Stomatology, Wenzhou Medical University, Wenzhou, China; ^8^ Department of Chemical and Materials Engineering, National Central University, Taoyuan, Taiwan; ^9^ Department of Chemical Engineering and R&D Center for Membrane Technology, Chung Yuan Christian University, Taoyuan, Taiwan

**Keywords:** dental pulp stem cells, synthetic cell culture polymer, neuronal cell differentiation, poly-L-ornithine, poly-N-isopropylacrylamide-butyl acrylate, cell therapy

## Abstract

Stem cells serve as an ideal source of tissue regeneration therapy because of their high stemness properties and regenerative activities. Mesenchymal stem cells (MSCs) are considered an excellent source of stem cell therapy because MSCs can be easily obtained without ethical concern and can differentiate into most types of cells in the human body. We prepared cell culture materials combined with synthetic polymeric materials of poly-N-isopropylacrylamide-co-butyl acrylate (PN) and extracellular matrix proteins to investigate the effect of cell culture biomaterials on the differentiation of dental pulp stem cells (DPSCs) into neuronal cells. The DPSCs cultured on poly-L-ornithine (PLO)-coated (TPS-PLO) plates and PLO and PN-coated (TPS-PLO-PN) plates showed excellent neuronal marker (*β*III-tubulin and nestin) expression and the highest expansion rate among the culture plates investigated in this study. This result suggests that the TPS-PLO and TPS-PN-PLO plates maintained stable DPSCs proliferation and had good capabilities of differentiating into neuronal cells. TPS-PLO and TPS-PN-PLO plates may have high potentials as cell culture biomaterials for the differentiation of MSCs into several neural cells, such as cells in the central nervous system, retinal cells, retinal organoids and oligodendrocytes, which will expand the sources of cells for stem cell therapies in the future.

## Introduction

Stem cells are considered as an ideal source of tissue regeneration therapy because of their stemness properties and regenerative abilities. Comparing embryonic stem cells (ESCs) and induced pluripotent stem cells (iPSCs), mesenchymal stem cells (MSCs) are an attractive source of stem cell therapy that does not involve ethical concerns or tumorigenesis possibilities. MSCs, which are typically isolated from bone marrow and fat tissues, have a good ability to differentiate into adipocytes, osteoblasts, and chondrocytes (Belka et al., 2019; Higuchi et al., 2019; Tsai et al., 2019; Albashari et al., 2020; Pan et al., 2020; Tan et al., 2020; Xing et al., 2020; Yang J. et al., 2020; El-Rashidy et al., 2021; Mohammed et al., 2021; Rozila et al., 2021) and show an ability to differentiate into not only these three types of cells but also ectoderm- or endoderm-derived cells (Georgiou et al., 2015; Andrzejewska et al., 2019; Han et al., 2021; He et al., 2020; Luo et al., 2021a; Luo et al., 2021b; Van Rensburg et al., 2021; Wu et al., 2021; Zhu et al., 2021a). For example, 5-azacytidine in cell culture medium induces MSC differentiation into cardiomyocytes and myoblasts (Xu et al., 2004). The induction of MSCs into hepatocytes can be achieved by stimulation with basic fibroblast growth factor (bFGF), epidermal growth factor (EGF) and nicotinamide, followed by additions of oncostatin M, dexamethasone, insulin, transferrin and selenium (Lee et al., 2004). MSCs can differentiate into neuronal cells by using *β*-mercaptoethanol, insulin, retinoic acid, bFGF, EGF, valproic acid, and hydrocortisol (Anghileri et al., 2008; Pavlova et al., 2012). Most previous studies focused on the development of differentiation medium to induce MSCs into cells derived from three different germ layers (Lee et al., 2004; Xu et al., 2004; Anghileri et al., 2008; Pavlova et al., 2012; Andrzejewska et al., 2019; Higuchi et al., 2019), and these studies demonstrated that MSCs had great potential to differentiate into cells derived from all three germ layers (Lee et al., 2004; Xu et al., 2004; Anghileri et al., 2008; Pavlova et al., 2012; Andrzejewska et al., 2019; Higuchi et al., 2019); in some ways, these characteristics are comparable to hPSCs (Higuchi et al., 2015; Andrzejewska et al., 2019). Currently, only a few studies have investigated the effect of cell culture biomaterials on MSC differentiation into specific lineages of cells, especially neural cell lineages (Prabhakaran et al., 2009; Zhang et al., 2016; Ghorbani et al., 2018; Luo et al., 2018a; Luo et al., 2018b; Hsiao et al., 2020; Luo et al., 2020; Albashari et al., 2021; Zhu et al., 2021b).

Prabhakaran et al. investigated hMSC differentiation into neurons on poly (L-lactic acid)-co-poly (3-caprolactone)/collagen (PLCL/Coll) nanofibrous scaffolds fabricated by using an electron spinning method (Prabhakaran et al., 2009). They found that the cell proliferation ratio of hMSCs cultured on PLCL/Coll nanofibrous scaffolds was the highest among the cell culture biomaterials examined in this study. An 80% higher cell proliferation was observed for the cells on the PLCL/Coll nanofibrous scaffolds than the cells cultured on PLCL nanofibrous scaffolds only (Prabhakaran et al., 2009). Although the cell proliferation on the PLCL/Coll nanofibrous scaffolds was slightly higher than that cultured on tissue culture polystyrene (TPS) plates, it is suggested that the combination of synthetic material (PLCL) and extracellular matrix (ECM) protein (Coll) can provide a balance with a stable cell proliferation rate and efficient differentiation abilities.

Ghorbani et al. investigated human Wharton jelly-derived mesenchymal stem cells (WJ-hMSCs) cultured on polylactic acid (PLA) scaffolds by using a wet-electrospinning method and by subsequently coating with alginate and gelatin, and the WJ-hMSCs were differentiated into neurons (Ghorbani et al., 2018). WJ-hMSCs cultured on the PLA scaffolds were more favored to differentiate into neuronal cells than those cultured on TPS plates, which was validated by immunostaining and qPCR assays of nestin, microtubule-associated protein 2 (MAP2), and neuron-specific enolase (NSE) expression (Ghorbani et al., 2018).

In our previous study (Sung et al., 2021a), human adipose-derived stem cells (hADSCs) were cultivated sequentially in a two-dimensional (2D) biomaterial surface and in a three-dimensional (3D) culturing condition. hADSCs cultured in 3D culture conditions showed higher expression of differentiation markers of osteogenic cells and chondrogenic cells than that of hADSCs cultured in 2D culture conditions when hADSCs were differentiated into osteogenic cells and chondrogenic cells (Sung et al., 2021a). However, the pluripotency and differentiation ability of hADSCs were extremely decreased when they were transferred from 3D culture conditions to 2D culture conditions and vice versa, which suggested that hADSCs have reversible characteristics in terms of their pluripotent characteristics and differentiation potential that depend on their environmental niche in 3D and 2D cultures.

In another study (Gao et al., 2019), hADSCs were cultured on several ECM-coated plates in xeno-free medium with human platelet lysate (hPL). Matrigel-coated surfaces suppressed hADSC differentiation into chondrocytes and facilitated hADSC induction into osteoblasts in media supplemented with 10% hPL (Gao et al., 2019). Fibronectin-coated and recombinant vitronectin-coated surfaces extensively facilitated hADSC induction into chondrocytes and osteoblasts in media supplemented with 5 and 10% hPL (Gao et al., 2019). hPL facilitated hADSC induction into chondrogenic and osteogenic differentiation compared to that of fetal bovine serum (FBS) on all tested ECM-immobilized surfaces.

In this study, we investigated the effect of cell cultivation biomaterials on the differentiation of dental pulp stem cells (DPSCs) into neuronal cells. DPSCs are categorized as one type of MSC. The isolation of DPSCs from a patient is a simple and easy process that does not involve pain or complex surgery. DPSCs can also be obtained from an infant’s tooth as the tooth changes into a permanent tooth. DPSCs show strong MSC markers of CD44, CD73, CD90 and CD105 on their surfaces (Machado et al., 2016; Luzuriaga et al., 2019; Noda et al., 2019). DPSCs and MSCs also share several characteristics. DPSCs belong to the neuronal crest; therefore, DPSCs have a greater tendency to secrete nerve growth factors (NGFs) and to differentiate into neuronal cells than that of bone marrow stem cells. Therefore, DPSCs should be an ideal cell source for neuronal repair in tissue regeneration therapy (d'Aquino et al., 2009; Pisciotta et al., 2015; Bonaventura et al., 2020; Pisciotta et al., 2020).

Suitable polymeric scaffolds can efficiently deliver therapeutic cells to the target site of injuries. Therefore, Feng et al. developed small 3D porous chitosan scaffolds using a freeze-drying process, and they demonstrated that the chitosan scaffolds can promote DPSCs differentiation into neuronal cells *in vitro* (Feng et al., 2014). They found that the DPSCs in the chitosan scaffolds expressed high expression of nestin, which was reduced sharply following differentiation (Feng et al., 2014). This investigation showed that granular 3D chitosan scaffolds provided a conducive and appropriate microenvironment for cell attachment, proliferation, and neuronal differentiation of DPSCs.

Hsiao et al. reported that DPSCs could successfully adhere to 3D-printed polylactic acid scaffolds (3DP-PLAS) modified by poly-L-lysine and demonstrated morphological changes and related protein expression in DPSCs (Hsiao et al., 2020). They also found that cellular orientations were more easily induced with DPSCs cultured on 3DP-PLAS with 150 μm gaps than with DPSCs cultured on 200 μm gaps. This study suggested that DPSCs cultured on 3DP-PLASs with narrow gaps in width showed better efficiency to differentiate into neurons (Hsiao et al., 2020).

Zhang et al. reported that DPSCs differentiated into neurons on chitosan scaffolds with an average pore diameter of 270 μm and showed the highest neuron marker expression (Zhang et al., 2016). The secretion of NT-3, b-NGF, GDNF and BDNF was significantly increased in the DPSCs/chitosan-scaffold group by 2.3-fold, 4.1-fold, 1.5-fold and 2.2-fold compared with the control group (DPSCs culture on TPS dishes), respectively. In a spinal cord injury (SCI) mouse model, the transplantation of DPSCs-derived neurons on chitosan scaffolds into mice significantly inhibited active caspase-3 (decrease in apoptotic cells) compared with that in the control groups (Zhang et al., 2016). DPSCs cultured on the scaffolds showed not only increased DPSCs-derived neuronal differentiation ability but also elevated efficacy in inhibiting SCI neuronal cell apoptosis after transplantation.

Zheng et al. performed DPSCs culture on chitosan scaffolds, which induced differentiation into neuronal cells for 7 days (Zheng et al., 2021). Among the comparison of DPSCs cultured on the control and DPSCs/chitosan-scaffold groups, the expression of glial fibrillary acidic protein (GFAP), S100*β* (astrocyte marker) and *β*Ⅲ-tubulin (neuronal marker) was extensively enhanced in the DPSCs/chitosan-scaffold group. Based on their findings (Zheng et al., 2021), chitosan scaffolds, which were not cytotoxic to the survival of DPSCs, were more favored for DPSCs neural differentiation.

MSCs typically reside in and are surrounded by extracellular matrix (ECM) proteins. ECM proteins surrounded by DPSCs *in vivo* typically contain collagen type IV, collagen type III and fibronectin with small amounts of collagen type I (Linde, 1985; Malara et al., 2014). Several glycoproteins, such as proteoglycans, hyaluronic acids and glycosaminoglycans, are also included in the DPSCs niche (Linde, 1985; Malara et al., 2014). These compositions are significantly different depending on the MSCs (Laudani et al., 2020). Therefore, there should be an optimal ECM protein that is suitable for DPSCs differentiation into neuronal cells. However, there is no research to investigate the effect of several ECM proteins as cell culture biomaterials on the differentiation of human DPSCs into neural cells from our database studies, although there are several studies (Arthur et al., 2008; Ebrahimi et al., 2011; Zainal Ariffin et al., 2013; Chang et al., 2014; Feng et al., 2014; Kanafi et al., 2014; Al-Zer et al., 2015; Mattei et al., 2015; Cho et al., 2016; Young et al., 2016; Zhang et al., 2016; Geng et al., 2017; Sanen et al., 2017; Ullah et al., 2017; Zhang et al., 2017; Ganapathy et al., 2019; Hsiao et al., 2020; Rafiee et al., 2020; Solis-Castro et al., 2020; Arimura et al., 2021; Darvishi et al., 2021; Zheng et al., 2021) that address human DPSCs differentiation into neural cells.

In this study, we screened ECM proteins together with synthetic polymeric materials of poly-N-isopropylacrylamide-butyl acrylate (PN), which has high potential to induce DPSCs into neuronal cells. We first evaluated DPSCs growth on PN-coated plates with and without ECM protein coating to achieve the best growth rate of DPSCs. Subsequently, we evaluated DPSCs differentiation into neuronal cells based on the neural marker expression of nestin (neural stem/progenitor cell marker) and *β*III-tubulin on PN-coated plates with and without ECM protein coating (Higuchi et al., 2019). We investigated the best composition of the PN and ECM proteins coating materials for DPSCs culture and differentiation into neuronal cells. This research has the potential to help develop optimal conditions for the differentiation of DPSCs into neuronal cells, which may be applied for future regenerative medicine for several neural diseases, such as age-related macular degeneration, spinal cord injury or Parkinson’s disease.

## Material and Methods

### Materials

The proteins, chemicals and biomaterials used in this research are summarized in Table 1. The other chemicals utilized in this project were received from Sigma-Aldrich (St. Louis, MO, United States).

**TABLE 1:** Materials used in this study.

Materials	Abbreviations	Cat. No.	Company
ECM			
Recombinant vitronectin	rVT	A14700	Thermo Fisher Scientific Inc. (Waltham, MA, United States)
Biolaminin 521	LN	BLA-LN521-05	BioLamina (Tokyo, Japan)
Poly-L-Ornithine	PLO	A-004-C	Merck (Darmstadt, Germany)
Chemicals and polymers			
Phosphate-buffered saline	PBS	c20012500BT	Thermo Fisher Scientific Inc. (Waltham, MA, United States)
Penicillin-Streptomycin	PS	15140122	Thermo Fisher Scientific Inc. (Waltham, MA, United States)
Dulbecco’s phosphate-buffered saline	DPBS	14040141	Thermo Fisher Scientific Inc. (Waltham, MA, United States)
Trypsin-EDTA (0.25%) solution	Trypsin-EDTA	25200-072	Thermo Fisher Scientific Inc. (Waltham, MA, United States)
Culture medium and component			
*α*-MEM medium	α-MEM	12571048	Thermo Fisher Scientific Inc. (Waltham, MA, United States)
Fetal bovine serum	FBS	10091148	Thermo Fisher Scientific Inc. (Waltham, MA, United States)
Neurobasal-A medium	Neurobasal-A	10888022	Thermo Fisher Scientific Inc. (Waltham, MA, United States)
Human recombinant basic fibroblast growth factor	bFGF	AF-100-18B	Peprotech (Rocky Hill, NJ, United States)
Human epidermal growth factor	EGF	AF-100-15	Peprotech (Rocky Hill, NJ, United States)
B27 supplement	B27	17504044	Thermo Fisher Scientific Inc. (Waltham, MA, United States)
Poly (*N*-isopropylacrylamide-co-butylacrylate)	PN	762881	Sigma-Aldrich (St. Louis, MO, United States)
Antibodies			
CD73 monoclonal antibody, FITC	CD 73	11-0739-41	Thermo Fisher Scientific Inc. (Waltham, MA, United States)
CD105 monoclonal antibody, PE	CD 105	12-1057-42	Thermo Fisher Scientific Inc. (Waltham, MA, United States)
CD34 monoclonal antibody, FITC	CD34	11-034941	Thermo Fisher Scientific Inc. (Waltham, MA, United States)
Mouse IgG1 kappa isotype, FITC	IgG1 isotype-FITC	11-471482	Thermo Fisher Scientific Inc. (Waltham, MA, United States)
Mouse IgG1 kappa Isotype, PE	IgG1 isotype-PE	12-4714-82	Thermo Fisher Scientific Inc. (Waltham, MA, United States)
Anti-nestin antibody	Nestin antibody	ab176571	Abcam (Milton, Cambridge, United Kingdom)
Anti-beta III tubulin antibody	*β*III tubullin antibody	ab78078	Abcam (Milton, Cambridge, United Kingdom)
Goat anti-rabbit IgG H&L antibody (AlexaFluor® 488)	488-Goat anti-rabbit IgG H&L antibody	ab150077	Abcam (Milton, Cambridge, United Kingdom)
Goat anti-mouse IgG H&L antibody (Alexa Fluor® 555)	555-Goat anti-mouse IgG H&L antibody	ab150114	Abcam (Milton, Cambridge, United Kingdom)

### Dental Pulp Stem Cell Extraction

**FIGURE 1:**
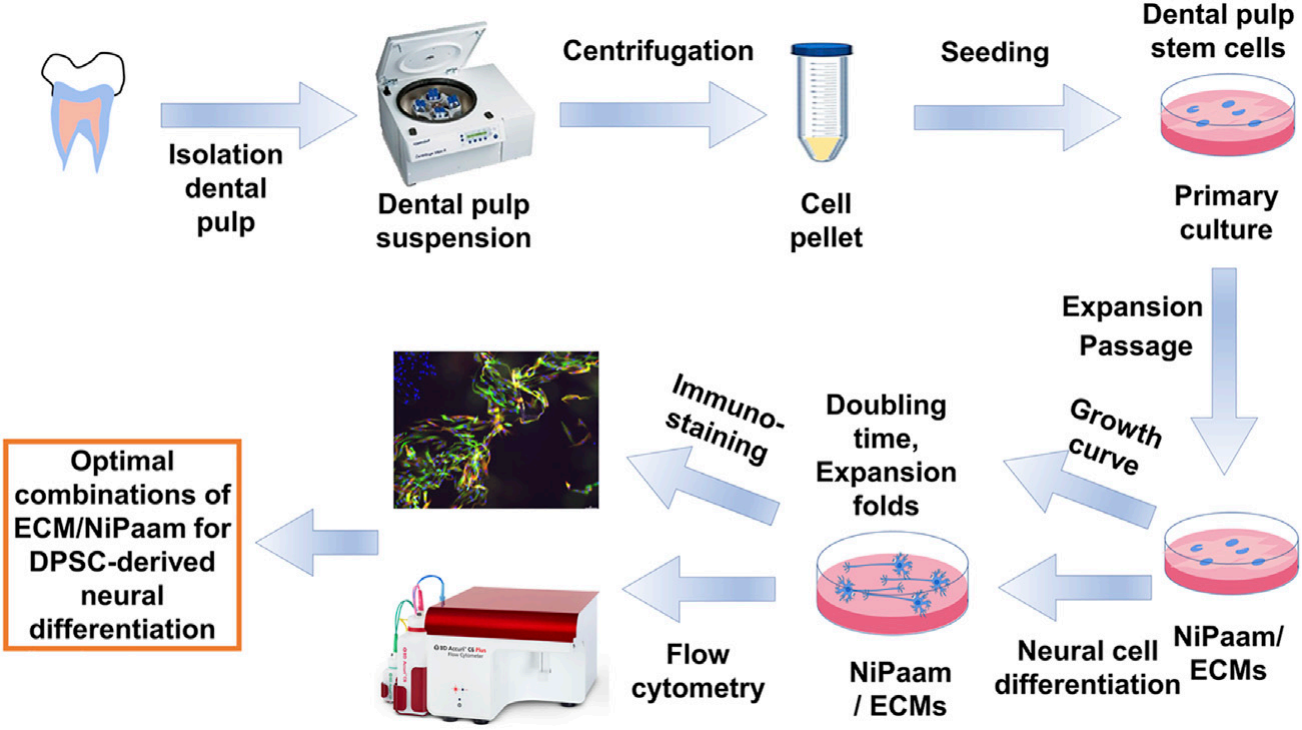
Experimental scheme of DPSCs extraction, culture, characterization and differentiation into neuronal cells.

Dental pulp was isolated from teeth after tooth extraction surgery with informed patient consent (10 patients, 5–18 years old). These informed consents were also acquired the agreement from a parent and/or legal guardian for this study. A schematic of DPSCs extraction from dental pulp is illustrated in Figure 1. The tooth surface was sterilized with 2% streptomycin-penicillin (SP) for 5 min. Teeth were cut and the inner dental pulp was exposed by using a surgical drill, scissors and knives. Dental pulp tissue was washed and suspended in Dulbecco’s modified Eagle’s medium (DMEM) supplemented with 10% FBS and 1% SP. Subsequently, the dental pulp tissue was seeded on TPS plates and incubated at 37°C under a 5% CO_2_ atmosphere. After the cells (DPSCs) reached 78–82% confluence, DPSCs were detached using a 0.25% trypsin-ethylenediaminetetraacetic acid (EDTA) solution by digestion, centrifuged and passaged into new TPS plates according to a conventional passage procedure. DPSCs at passages 5-7 were used for the following experiments.

### Preparation of Cell Culture Biomaterials

TPS-PN plates (poly-N-isopropylacrylamide-co**-**butyl acrylate-coated TPS plates) were prepared as follows. Poly-N-isopropylacrylamide-co**-**butyl acrylate, PN, which has a lower critical solution temperature (LCST) of approximately 30°C with a molecular weight of 88,000, was dissolved to 1.8 mg/ml in 99.5% ethanol. The PN solution was inserted into TPS 6-well plates (1.5 ml per well, surface area of 9.6 cm^2^) and incubated at 37°C for 2 h. Then, the plates were rinsed using 1 ml of 99.5% ethanol per well four times, and then the plates were rinsed with 1 ml of ultrapure water per well four times. Subsequently, the plates were rinsed with 1 ml of 5, 3 and 1% antibiotic-antimycotic solution per well sequentially.

Laminin-521 (LN), poly-L-ornithine (PLO), and recombinant vitronectin (rVT) were prepared as 5 μg/ml solutions utilizing phosphate buffered solution (PBS). LN, PLO, or rVT solution was inserted into 6-well plates in which PN was either coated (TPS-PN) in advance or not coated (TPS), and the plates were incubated at 37°C for 2 h. Then, the LN, PLO, or rVT solution was removed from 6-well plates, and the plates were utilized for subsequent DPSCs cultivation and differentiation experiments. TPS-LN, TPS-rVT and TPS-PLO plates indicate LN-coated TPS, rVT-coated TPS and PLO-coated TPS plates, respectively. TPS-PN-LN, TPS-PN-rVT and TPS-PN-PLO indicate LN-coated TPS-PN, rVT-coated TPS-PN and PLO-coated TPS-PN plates, respectively.

### Characterization of the Cell Culturing Plates

Chemical analysis (N1s and C1s) of TPS and the TPS-PN, TPS-ECM (ECM = rVT, LN or PLO) and TPS-PN-ECM plates were performed using XPS (X-ray photoelectron spectroscopy, Thermal Scientific, Inc., Amarillo, TX, United States) as previously described (Sung et al., 2021b). The binding energy scale adjustment was set from the peak maximum at 284.6 eV in the C1s spectrum.

### DPSCs Characterization

Doubling time of DPSCs was calculated from following formula:

$$\text{Doubling}\;\text{time}=X\text{∗Ln}\left(2\right)/\text{Ln}\left(N_{\text{X}}/N_{\text{O}}\right)$$ (1)

where *N*_X_ indicates cell number at *X* day and *N*_O_ indicates the initial cell number.

CD34 (negative marker, hematopoietic stem cell and endothelial progenitor marker), CD73 (MSC marker), and CD105 (MSC marker) expression in DPSCs was evaluated using flow cytometry (BD Accuri™ C6, BD Biosciences, Franklin Lakes, NJ, United States) (Figure 1). The cells were incubated with anti-CD markers (1:500 dilution) or isotype antibodies (1:500 dilution) for 45 min, and the solution was centrifuged at 450 × g for 8 min. The cells were transferred into a phosphate-buffered saline solution and evaluated utilizing flow cytometry as previously described (Sung et al., 2021a).

### Inducing the Differentiation of DPSCs Into Neuronal Cell

The procedure of inducing the differentiation of DPSCs into neuronal cells was followed by the method reported by Wang et al. with some modifications [12]. On day −5, DPSCs were inoculated into ECM- and/or PN-coated plates and cultured in DPSCs medium (DMEM supplemented with 10% FBS and 1% SP) until day 0 by exchanging the DPSCs medium every other day. On day 0, DPSCs displayed approximately 85–90% confluence. The DPSCs cultivation medium was discarded, and neurobasal-A medium supplemented with 2 wt% B27, 20 ng/ml EGF and 20 ng/ml bFGF was added to the plates. The media were changed every 3 days. The cells were cultivated until day 15. We investigated whether the purity of DPSCs-derived neuronal cells was facilitated by different cell culture surfaces. These cells were analyzed for the expression levels of *β*III-tubulin and nestin using flow cytometry and immunostaining.

### Statistical Analysis

Experimental results were analyzed from four replicates. The data are shown as the mean ± standard deviation (SD). Statistical analyses were processed using One-way ANOVA in Excel (Microsoft Corporation) with post-hoc *t*-test. Probability values (*p*) less than 0.05 were considered statistically significant.

## Results

### XPS Assay of PN-Coated and PN Plus ECM-Coated Plates

We developed PN-coated (TPS-PN) and PN plus ECM-coated plates (TPS-PN-ECM) as well as ECM-coated (TPS-ECM) plates for DPSCs culture and differentiation. Although cells can extensively attach to PN-coated plates more than to TPS plates, neuronal cells need to attach to the plates *via* specific integrin binding. Therefore, not only PN-coated plates but also PLO or ECM (rVT and LN) were further coated on PN-coated plates (TPS-PN-PLO, TPS-PN-rVT and TPS-PN-LN) in this study (Figure 1).

**FIGURE 2:**
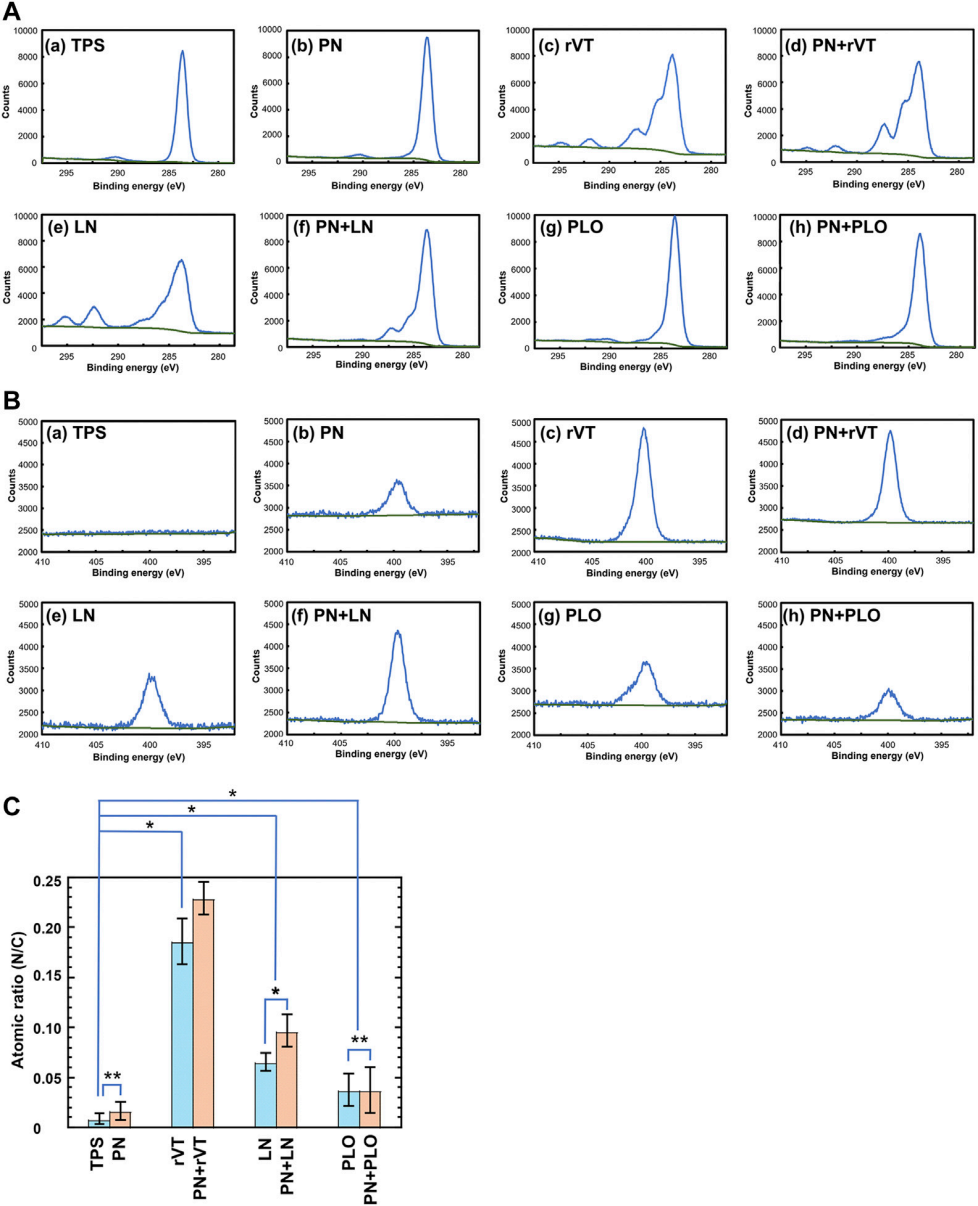
Characterization of TPS, TPS-PN, TPS-ECM, TPS-PLO, TPS-PN-ECM and TPS-PN-PLO plates. **(A)** High-resolution XPS spectra of the C1s peaks on the surface of TPS **(a)**, TPS-PN **(b)**, TPS-rVT **(c)**, TPS-PN-rVT **(d)**, TPS-LN **(e)**, TPS-PN-LN **(f)**, TPS-PLO **(g)** and TPS-PN-PLO **(h)** plates. **(B)** High-resolution XPS spectra of the N1s peaks on the surface of TPS **(a)**, TPS-PN **(b)**, TPS-rVT **(c)**, TPS-PN-rVT **(d)**, TPS-LN **(e)**, TPS-PN-LN **(f)**, TPS-PLO **(g)** and TPS-PN-PLO **(h)** plates. **(C)** The nitrogen to carbon (N/C) atomic ratios on the surface of TPS, TPS-PN, TPS-ECM, TPS-PLO, TPS-PN-ECM and TPS-PN-PLO plates. *p < 0.05. **p > 0.05.

These plates were analyzed to investigate the existence of PN and/or ECM on the plate surface utilizing XPS before the cultivation and differentiation of DPSCs into neuronal cells, where ECM-coated plates (TPS-rVT, TPS-LN, TPS-PLO, TPS-PN-rVT, TPS-PN-LN and TPS-PN-PLO) as well as TPS and TPS-PN plates were characterized. Figure 2 displays the high-resolution XPS spectra of the C1s (Figure 2A) and N1s (Figure 2B) peaks of the (a) TPS surface (negative control), (b) TPS-PN (PN-coated TPS) surface, (c) TPS-rVT (rVT-coated TPS) surface, (d) TPS-PN-rVT (rVT-coated TPS-PN) surface, (e) TPS-LN (LN-coated TPS) surface, (f) TPS-PN-LN (LN-coated TPS-PN) surface, (g) TPS-PLO (PLO-coated TPS) surface and (h) TPS-PN-PLO (PLO-coated TPS-PN) surface.

The C1s peak at approximately 285 eV was observed on any surface investigated in this study (Figure 2A). A relatively broad C1s peak was detected on the ECM-coated surface (TPS-rVT, TPS-PN-rVT, TPS-LN and TPS-PN-LN plates) compared to that on the TPS, TPS-PN, TPS-PLO and TPS-PN-PLO plates, which indirectly suggested the existence of ECM on ECM-coated plates.

The N1s peak at 400 eV was extensively observed on TPS-rVT, TPS-PN-rVT, TPS-LN, TPS-PN-LN, TPS-PLO, and TPS-PN- PLO plates, whereas no distinct N1s peak was found on the TPS surface, and a weak N1s peak was observed on the TPS-PN surface (Figure 2B). These findings were directly related to the atomic ratio of N/C described in Figure 2C and suggested the existence of ECM and PLO on ECM-coated and PLO-coated plates.

### DPSCs Extraction and Characterization

**FIGURE 3:**
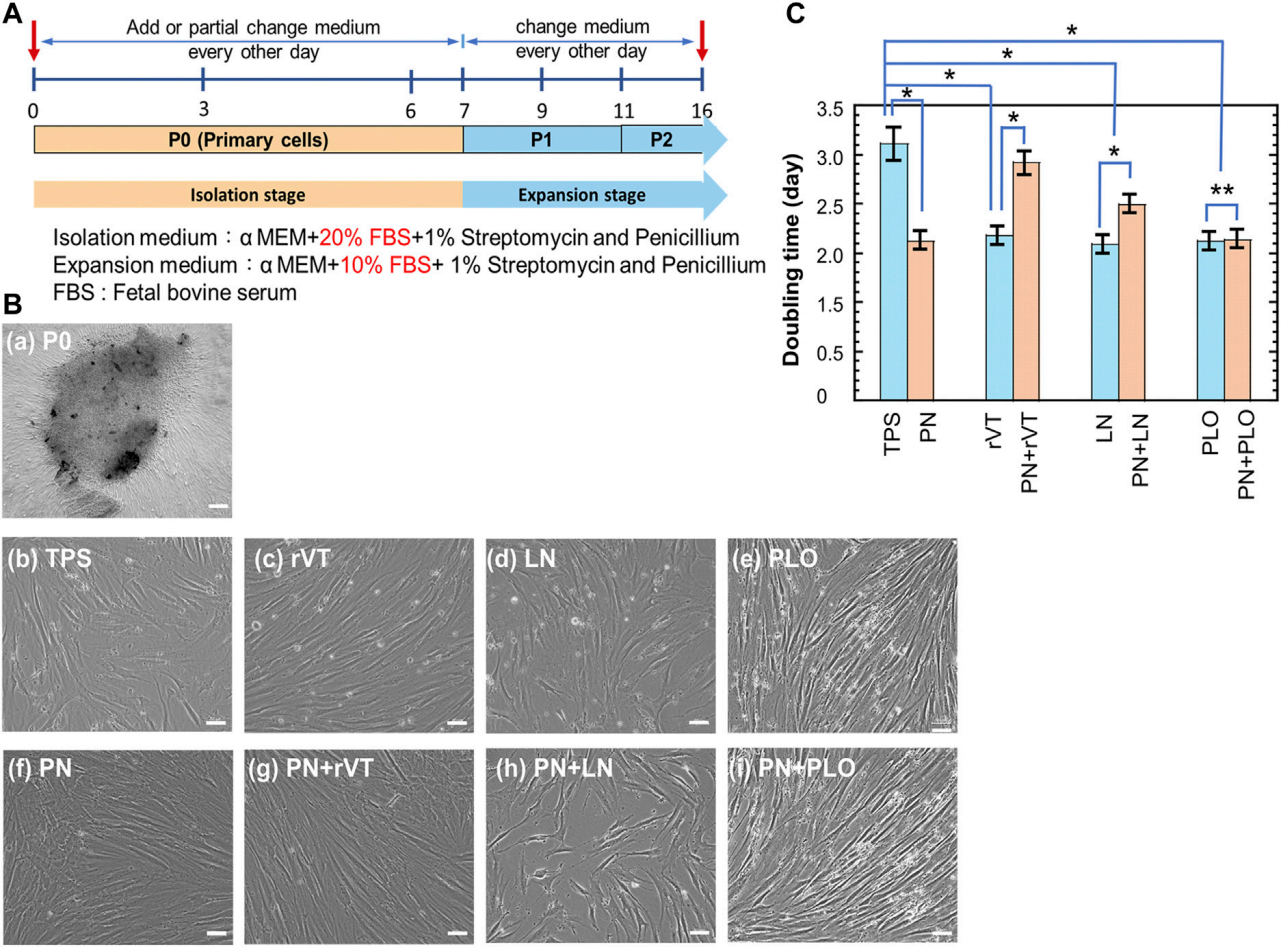
Cultivation of DPSCs on TPS, TPS-PN, TPS-ECM, TPS-PLO, TPS-PN-ECM and TPS-PN-PLO plates for five passages. **(A)** The timeline of primary DPSCs culturing and expansion. **(B)** The morphologies of primary DPSCs (day 5 at P0) **(a)** and day 5 at P5 **(b–i)** on the TPS **(b)**, TPS-rVT **(c)**, TPS-LN **(d)**, TPS-PLO **(e)**, TPS-PN **(f)**, TPS-PN-rVT **(g)**, TPS-PN-LN **(h)** and TPS-PN-PLO **(i)** plates. **(C)** The doubling time of DPSCs on TPS, TPS-PN, TPS-ECM, TPS-PLO, TPS-PN-ECM and TPS-PN-PLO plates at passages 3-5. The bar indicates 200 μm **(a)** and 50 μm **(b–i)**. *p < 0.05. **p > 0.05.

Dental pulp stem cells (DPSCs) were isolated from discarded teeth after tooth removal surgery. The process of DPSCs preparation is schematically described in Figure 1. The dental pulp was isolated from teeth, the surface of the dental pulp was sterilized with 2% SP solution, and the dental pulp was incised to expose the inner pulp. The inner pulp was suspended in medium and subsequently cut into 1–2 mm^2^ square small pieces of inner pulp tissue by using sterilized surgery scissors. Then, the pulp tissue was centrifuged to obtain inner pulp tissue pellets. Subsequently, the inner pulp tissues (dental pulp cells) were inserted into (a) TPS plates, (b) PN-coated (TPS-PN) plates, (c) ECM (rVT and LN)-coated (TPS-rVT and TPS-LN) plates, (d) PLO-coated (TPS-PLO) plates and (e) PN-coated plates where ECM (rVT and LN) or PLO was subsequently coated (TPS-PN-rVT, TPS-PN-LN and TPS-PN-PLO) plates. Subsequently, the cells were cultured on these plates for 10 passages in DMEM supplemented with 10% FBS (Figure 3A). After 1 week of cell cultivation, the cells migrated and extended from the pulp, which could be observed under microscopic observation. These cells were considered to be primary DPSCs (passage 0, P0) (Figure 3B). When DPSCs became confluent on the plates, DPSCs at P0 were detached by using a 0.25% trypsin-EDTA digestion for the passage process. After performing a passage and subsequent cell culture for 14 days, the cells started to show more spindle morphologies, which are characteristics of MSCs (DPSCs) (Figure 3B). These primary cells were cultured for 5 passages (P5).

We evaluated the doubling time of the cells, which were cultured on different cell culture biomaterials, during passages 3-5 and the results are shown in Figure 3C. The fastest DPSC doubling time was 2.1 days, in which DPSCs were cultured on TPS-PN, TPS-rVT, TPS-LN, TPS-PLO and TPS-PN-PLO plates. Compared with the DPSCs on TPS plates (doubling time = 3.1 days), DPSCs on TPS-PN, TPS-rVT, TPS-LN, TPS-PLO and TPS-PN-PLO plates (doubling time = 2.1 days) had a doubling time that was faster by 1 day (*p* < 0.05). In the other ECM-coated plate group, the doubling times of DPSCs on TPS-PN-rVT and TPS-PN-LN plates were 2.9 and 2.5 days, respectively, which were 0.2 and 0.6 days faster than the cells cultured on TPS plates, respectively (*p* < 0.05 for TPS vs. TPS-PN-LN). This indicates that PN coating with ECMs causes a longer doubling time for DPSCs cultured on PN and ECM coating plates compared to that of the cells on the same ECM-coated plates without PN coatings (*p* < 0.05). However, the doubling time of DPSCs was not significantly different when the cells were cultured on TPS-PLO and TPS-PN-PLO plates (*p* > 0.05), in which the doubling time of DPSCs on these plates was the shortest (2.1 days) in this study. This result indicated that the TPS-PLO and TPS-PN-PLO plates were the most stable culturing materials for DPSCs cultivation and expansion.

### MSC Marker Expression of DPSCs Cultured on ECM and PN-Coated Plates

**FIGURE 4:**
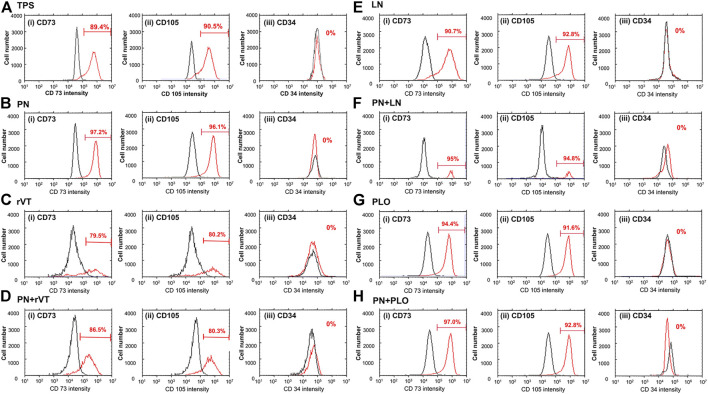
Expression of MSC markers [CD73 **(i)** and CD105 **(ii)**] and hematopoietic marker [CD34 **(iii)**] in DPSCs cultured on TPS **(A)**, TPS-PN **(B)**, TPS-rVT **(C)**, TPS-PN-rVT **(D)**, TPS-LN **(E)**, TPS-PN-LN **(F)**, TPS-PLO **(G)** and TPS-PN-PLO plates **(H)**.

We evaluated the MSC marker expression of CD 73 and CD 105 and hematopoietic marker expression of CD 34 on DPSCs cultured on (a) TPS plates, (b) PN-coated (TPS-PN) plates, (c) ECM-coated (TPS-rVT and TPS-LN) plates, (d) PLO-coated (TPS-PLO) plates and (e) PN-coated plates where ECM (TPS-PN-rVT and TPS-PN-LN) or PLO was subsequently coated (TPS-PN-PLO) were examined, and the results are shown in Figure 4. In general, all MSC markers of DPSCs cultured on different materials showed expressions of more than 90% except for DPSCs on TPS-rVT and TPS-PN-rVT plates, whereas CD34 was not expressed in the DPSCs cultured on any plates investigated in this study. DPSCs showed the lowest CD 73 and CD105 marker expression on TPS-rVT plates at 79.5 and 80.2%, respectively. The highest CD73 and CD105 expression was found for DPSCs cultured on TPS-PN-PLO plates, which was 97 and 92.8%, respectively. The difference among DPSCs cultured on ECM- or PLO-coated plates with or without PN coating were less distinct. These results suggested that the MSC marker expression of DPSCs was relatively high on any cell culture biomaterial investigated in this study. However, it should be noted that DPSCs cultured on TPS-PN-PLO plates showed the highest MSC surface marker expression, which may allow DPSCs to maintain their proliferation ability during the culturing process.

### Neuronal Cell Differentiation of DPSCs on ECM- and PN-Coated Plates

**FIGURE 5:**
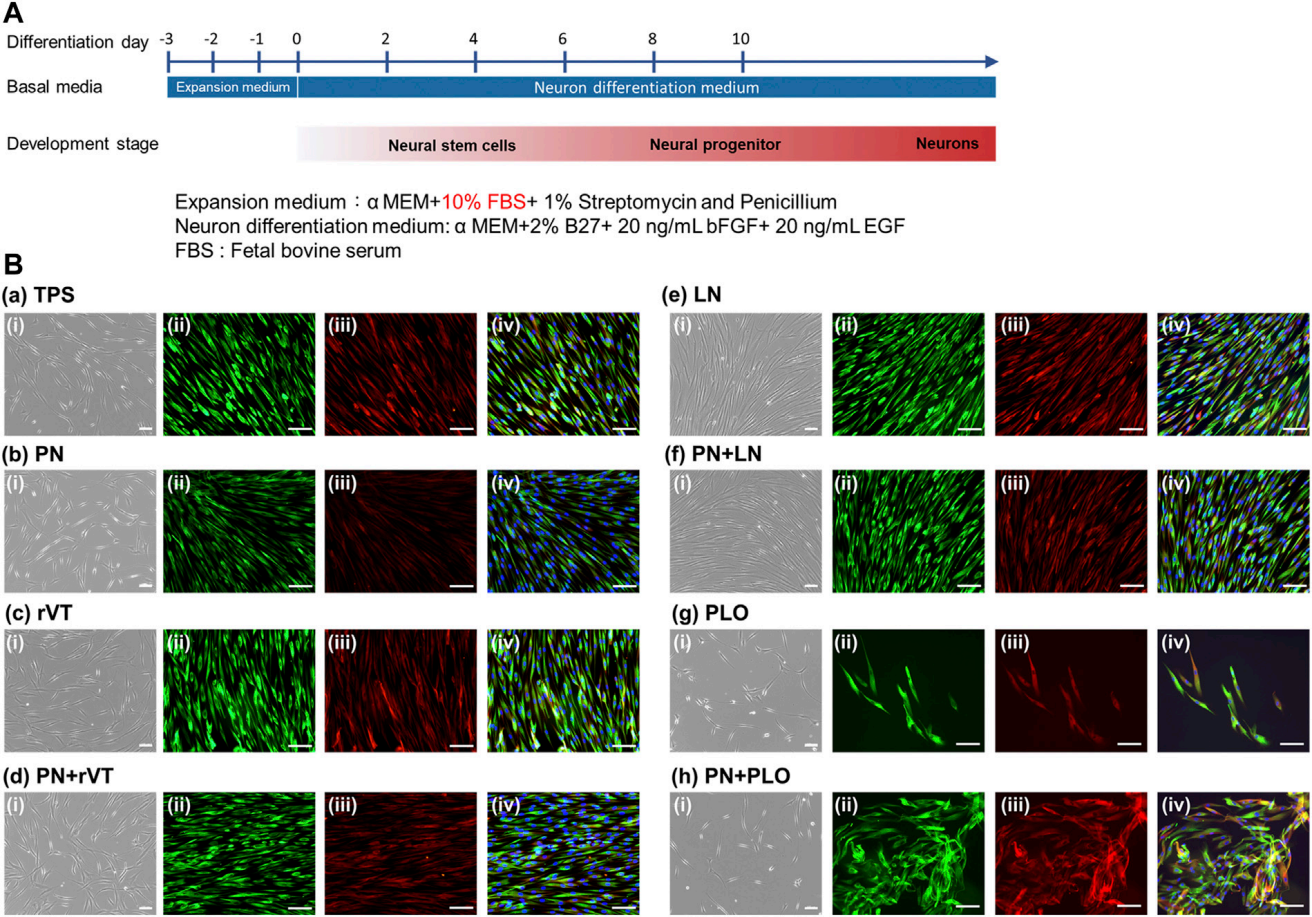
Differentiation of DPSCs into neuronal cells after five passages of cultivation on TPS, TPS-PN, TPS-ECM, TPS-PLO, TPS-PN-ECM and TPS-PN-PLO plates. **(A)** The timeline of the induction of DPSCs differentiation into neuronal cells. **(B)** The morphologies of DPSCs-derived neuronal cells **(i)** and the immunohistochemical assay of *β*III-tubulin [**(ii)**, green] and nestin [**(iii)**, red)] expression on the TPS **(a)**, TPS-PN **(b)**, TPS-rVT **(c)**, TPS-PN-rVT **(d)**, TPS-LN **(e)**, TPS-PN-LN **(f)**, TPS-PLO **(g)** and TPS-PN-PLO **(h)** plates. The photo **(iv)** was the merged photos of **(ii)** and **(iii)**. The scale bar indicates 100 μm.

DPSCs were induced into neuronal cell differentiation using neuron basal medium supplemented with B27, EGF and bFGF (Figure 5A), which were cultured on ECM-coated (TPS-LN and TPS-rVT) plates, PLO-coated (TPS-PLO) plates and PN-coated (TPS-PN, TPS-PN-rVT, TPS-PN-LN and TPS-PN-PLO) plates as well as noncoated TPS plates. Supplementary Figure S1 displays the sequential morphological changes in DPSCs induced into neuronal cells on ECM- and/or PN-coated plates. Extended long cell colonies began to gradually appear with increasing time after day 4. Several colonies detached from the edge as time passed, especially from the surface of ECM-coated plates, and then, the colonies of the dead cells peeled off from the plates. After day 8, we observed that the cells on each plate surface had neuronal fiber-like morphologies. The neuronal cell marker assay was further performed in the following experiments.

### Characterization of DPSCs-Derived Neuronal Cells on ECM and PN-Coated Plates

The expression of the neuronal cell marker proteins *β*III-tubulin and nestin on DPSCs-derived neuronal cells, which were cultured on PN-coated plates (TPS-PN, TPS-PN-rVT, TPS-PN-LN and TPS-PN-PLO), ECM-coated plates (TPS-rVT and TPS-LN), PLO-coated plates (TPS-PLO) and TPS plates, was evaluated using an immunofluorescence staining assay at day 15 of differentiation, and the results are shown in Figure 5B. DPSCs-derived neuronal cells, which could induce differentiation on all of the plates in this study, showed extensive expression of *β*III-tubulin and nestin. There was no significant difference in the expression of *β*III-tubulin and nestin in the cells cultivated on the TPS, TPS-LN and TPS-PN-LN plates or on the TPS-PN, TPS-rVT and TPS-PN-rVT plates. However, we found that the DPSCs differentiated on TPS-PN-PLO plates had the highest expression of *β*III-tubulin and nestin in the immunostaining assay in this study (Figure 5B).

**FIGURE 6:**
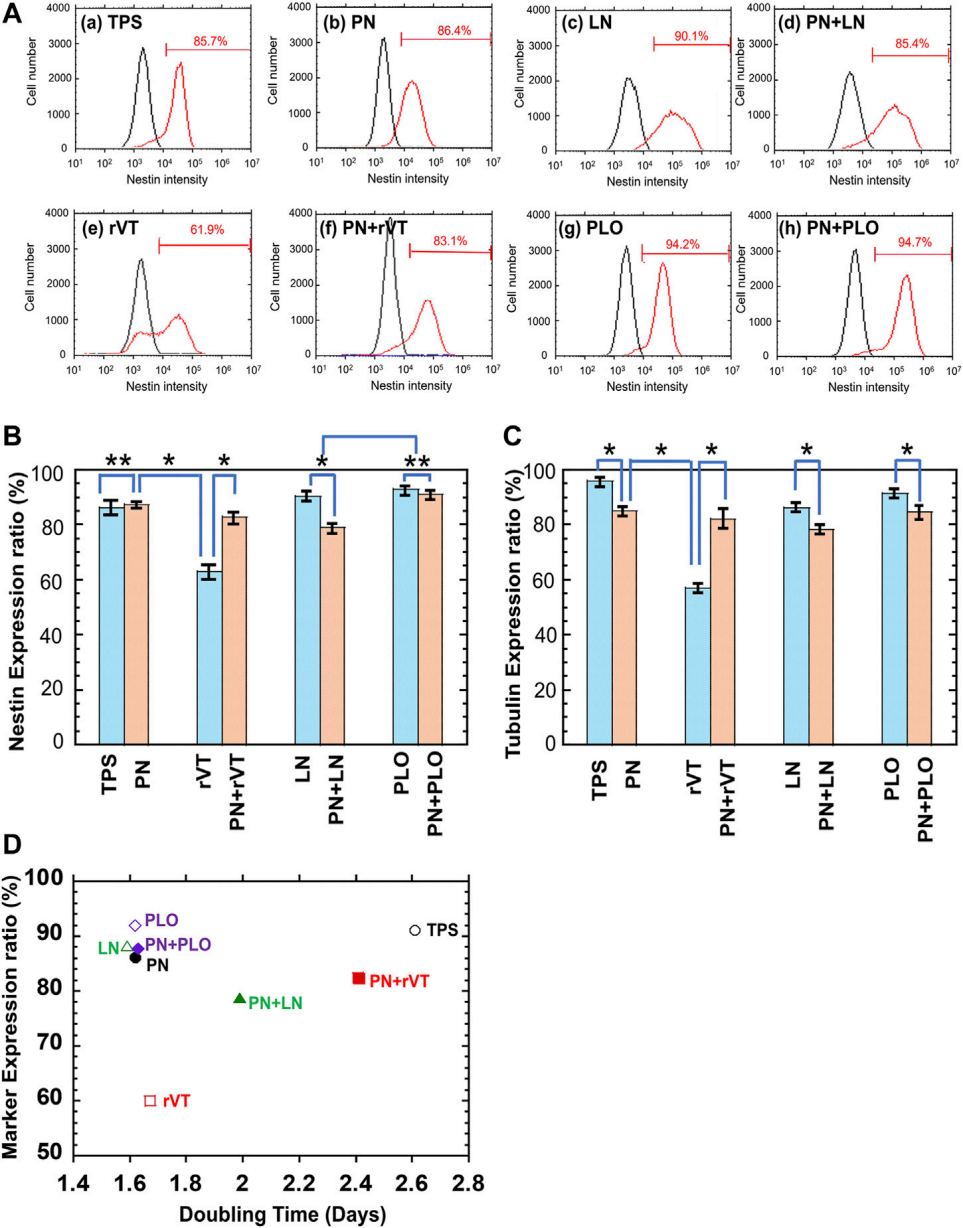
Characterization of DPSCs-derived neuronal cells after five passages of DPSCs cultivation on TPS, TPS-PN, TPS-ECM, TPS-PLO, TPS-PN-ECM and TPS-PN-PLO plates. **(A)** Flow cytometry spectra of nestin expression on DPSCs-derived neuronal cells cultivated on TPS **(a)**, TPS-PN **(b)**, TPS-LN **(c)**, TPS-PN-LN **(d)**, TPS-rVT **(e)**, TPS-PN-rVT **(f)**, TPS-PLO **(g)** and TPS-PN-PLO **(h)** plates. **(B)** Nestin expression ratio cultured on DPSCs-derived neuronal cells, which were cultivated on TPS, TPS-PN, TPS-ECM, TPS-PLO, TPS-PN-ECM and TPS-PN-PLO plates. *p < 0.05. **p > 0.05. **(C)** *β*III tubulin expression ratio cultured on DPSCs-derived neuronal cells, which were cultivated on TPS, TPS-PN, TPS-ECM, TPS-PLO, TPS-PN-ECM and TPS-PN-PLO plates. *p < 0.05. **(D)** Dependence of the averaged neuronal expression of DPSC-derived neuronal cells on the doubling time of DPSCs cultured on TPS, TPS-PN, TPS-ECM, TPS-PLO, TPS-PN-ECM and TPS-PN-PLO plates.

We also quantitatively evaluated whether the differentiation rate of DPSCs-derived neuronal cells was enhanced on the PN surface. After 15 days of differentiation, the neuronal marker (*β*III-tubulin and nestin) expression of DPSCs-derived neuronal cells was evaluated using flow cytometry. The flow cytometry charts of DPSCs-derived neuronal cells, which were cultivated on different culturing materials (rVT-, LN-, PLO-coated TPS dishes with or without PN coating surface as well as TPS and TPS-PN surface), are shown in Figure 6A for nestin expression and Supplementary Figure S2 for *β*III-tubulin expression. The averaged nestin and *β*III-tubulin expression of these cells is described in Figures 6B,C, respectively. DPSCs-derived neuronal cells cultured on TPS-PLO and TPS-PN-PLO plates showed more than 90% nestin expression and more than 85% *β*III-tubulin expression, which were comparable to that of the cells cultured on TPS-PN or TPS plates. Although DPSCs-derived neuronal cells exhibited high expression of *β*III-tubulin and nestin on the TPS plates, the expansion fold of DPSCs on TPS plates was significantly less (the doubling time was high) than that of the cells cultured on TPS-PLO and TPS-PN-PLO plates (Figure 6D).

The relationship between the neuronal marker expression rate (average rate of *β*III-tubulin and nestin expression) on DPSCs-derived neuronal cells and the fold expansion of DPSCs was investigated and plotted in Figure 6D. This plot indicated that the TPS-PN, TPS-PLO, TPS-PN-PLO and TPS-LN plates were the most suitable cell culture biomaterials for DPSC expansion and differentiation into neuronal cells.

## Discussion

DPSCs and DPSCs-derived neuronal cells were successfully cultured and differentiated on rVT-, LN-, and PLO-coated plates where PN was coated or not coated on the plates in advance. These ECM-coated and PLO-coated plates, which were combined with or without PN coating, could be easily prepared by the coating method. In our previous study (Peng et al., 2016; Yang L. et al., 2020), poly (N-isopropylacrylamide-co-styrene), PN, was used as a coating material for hESC and hiPSC cultivation. The current study indicated that PN-coated plates were not favorable for DPSCs culture and differentiation except for the TPS-PN and TPS-PN-PLO plates (Figure 6D). Neuronal cells derived from DPSCs on TPS-rVT plates showed less *β*III-tubulin and nestin expression than that of cells cultured on other the plates investigated in this study, such as the TPS-PN, TPS-PN-rVT, TPS-LN, TPS-PN-LN, TPS-PLO and TPS-PN-PLO plates. By the combination coating of rVT and PN on the plates, the cells on TPS-PN-rVT plates showed similar expression of *β*III-tubulin and nestin compared to that of the cells on TPS-PLO and TPS-PN-PLO plates. Therefore, PN coating on the plates is important for rVT-coated plates to enhance neuronal differentiation and the expansion of DPSCs. The cells on the TPS-PN-PLO plates showed similar expressions of *β*III-tubulin and nestin and similar expansion folds compared to that of the cells on PLO-TPS plates. The cells on TPS-LN plates also showed high expressions of *β*III-tubulin and nestin and had relatively good expansion folds. It should be noted that the cells cultured on TPS-PLO or TPS-PN-PLO plates showed a more than 20-fold expansion rate, which is the highest expansion rate in this study and 2-fold higher than that of the cells on TPS or TPS-PN plates. These results suggest that the DPSCs cultured on TPS-PLO and TPS-PN-PLO plates maintained their stable proliferation and good ability to differentiate to neuronal cells (Figures 3, 6). We expect our culture plates, such as the TPS-PLO and TPS-PN-PLO plates, to have high potential for being applied as cell culture biomaterials for the differentiation of MSCs into other neural cells, such as neural cells in the central nervous system, retinal cells (Smith et al., 2019; Michelet et al., 2020), retinal organoids (Cowan et al., 2020; O'Hara-Wright and Gonzalez-Cordero, 2020) and oligodendrocytes (Higuchi et al., 2019), to extend the sources of cells for stem cell therapies in the future.

## Data Availability

The raw data supporting the conclusion of this article will be made available by the authors, without undue reservation.
