# Supplementary material for: Neuronal Cell Differentiation of Human Dental Pulp Stem Cells on Synthetic Polymeric Surfaces Coated With ECM Proteins
Source: Front Cell Dev Biol. 2022 Jun 14;10:893241. doi: 10.3389/fcell.2022.893241 (PMC9237518; doi:10.3389/fcell.2022.893241)
Supplement: Supplementary file 1 [file DataSheet1.PDF]

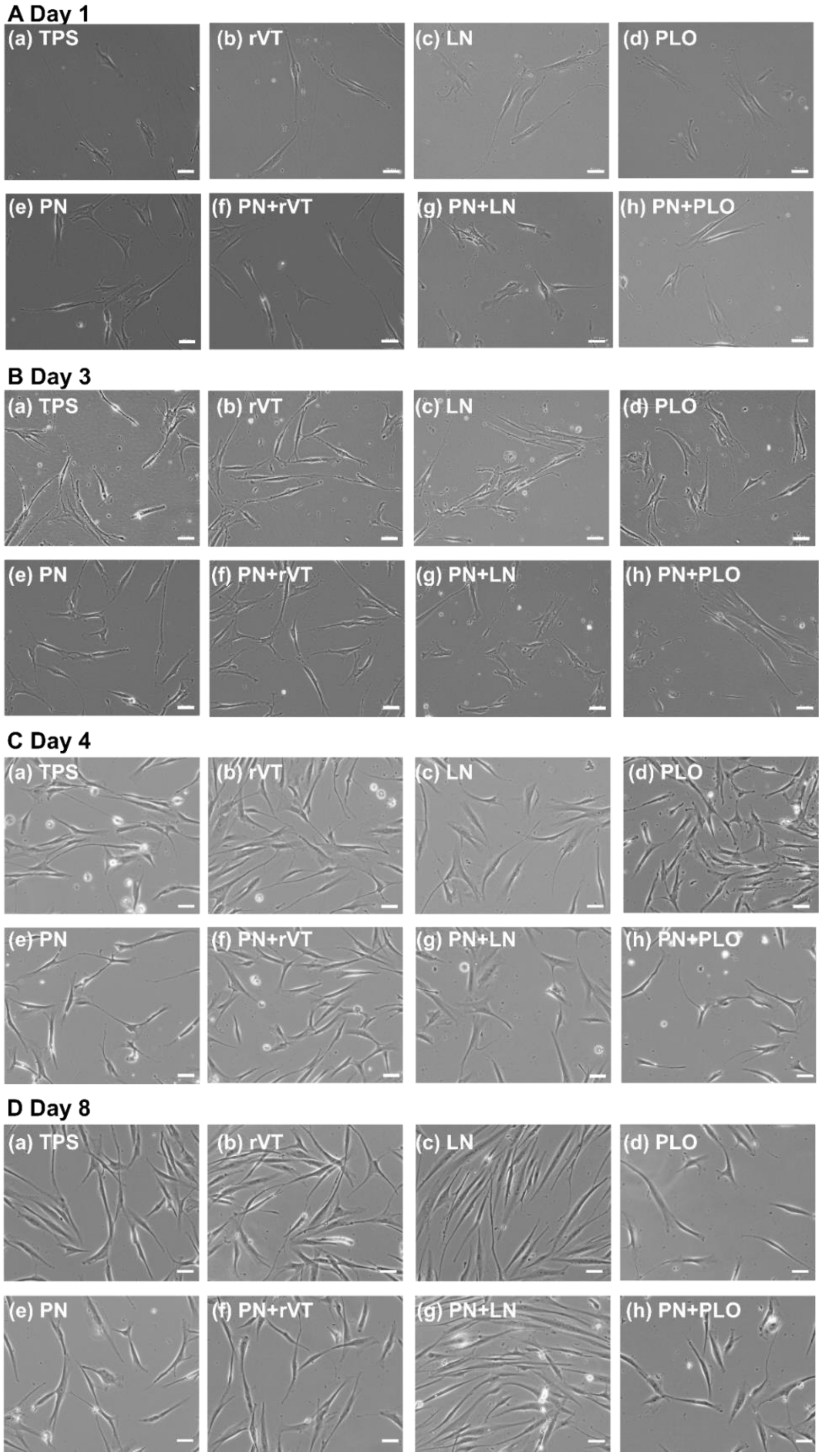

Supplementary Figure 1. Sequential morphological changes in the DPSCs differentiated into neuronal cells on TPS (a), TPS-rVT (b), TPS-LN (c), TPS-PLO (d), TPS-PN (e), TPS-PN-rVT (f), TPS-PN-LN (g) and TPS-PN-PLO (h) plates at day 1 (A), day 3 (3), day 4 (C) and day 8 (D).

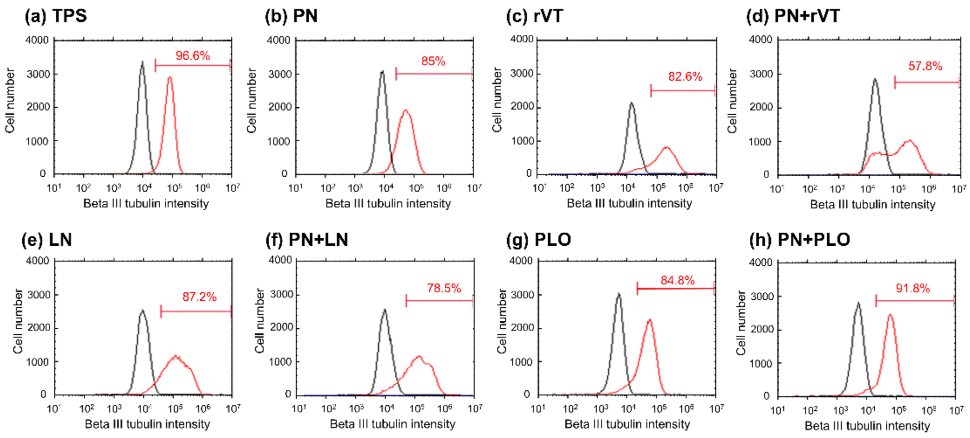

Supplementary Figure 2. Characterization of DPSC-derived neuronal cells after five passages of DPSC cultivation on TPS, TPS-PN, TPS-ECM, TPS-PLO, TPS-PN-ECM and TPS-PN-PLO plates. (A) Flow cytometry spectra of βIII-tubulin expression on DPSC-derived neuronal cells cultivated on TPS (a), TPS-PN (b), TPS-rVT (c), TPS-PN-rVT (d), TPS-LN (e), TPS-PN-LN (f), TPS-PLO (g) and TPS-PN-PLO (h) plates.
